# Pharmacognostic Study of the Leaves of *Ptilostemon greuteri* Raimondo & Domina, a Rare Sicilian Paleoendemic Species

**DOI:** 10.3390/plants14030370

**Published:** 2025-01-26

**Authors:** Antonella Smeriglio, Domenico Trombetta, Laura Cornara, Paola Malaspina, Mariarosaria Ingegneri, Emilio Di Gristina, Enrico Bajona, Flavio Polito, Vincenzo De Feo

**Affiliations:** 1 Department of Chemical, Biological, Pharmaceutical and Environmental Sciences (ChiBioFarAm), University of Messina, 98166 Messina, Italy; antonella.smeriglio@unime.it (A.S.); domenico.trombetta@unime.it (D.T.); mariarosaria.ingegneri@unime.it (M.I.); 2 Department of Earth, Environment and Life Sciences, University of Genova, Corso Europa 26, 16132 Genova, Italy; paola.malaspina@unige.it; 3 Department of Agricultural, Food and Forest Sciences (SAAF), University of Palermo, 90128 Palermo, Italy; emilio.digristina@unipa.it (E.D.G.); enrico.bajona@unipa.it (E.B.); 4 Department of Pharmacy, University of Salerno, Via Giovanni Paolo II 132, 84084 Fisciano, Italy; fpolito@unisa.it (F.P.); defeo@unisa.it (V.D.F.)

**Keywords:** Asteraceae, micro-morphology, phytochemistry, fatty acids, terpenes, polyphenols, antioxidant activity, anti-inflammatory activity

## Abstract

*Ptilostemon greuteri* Raimondo & Domina is a rare Sicilian paleoendemic species. The aim of study was to investigate the micro-morphological features of leaves by light and scanning electron microscopy, to elucidate the phytochemical profile of essential oil (EO), *n*-hexane (HE) and hydroalcoholic extract (HAE) by gas and liquid chromatographic methods, and antioxidant and anti-inflammatory properties by in vitro assays. Leaves had a large lanceolate blade, dark green on the upper side and greyish on the lower one with a dense tomentum. Epidermis showed many protruding stomata. By lipid-specific dyes, lipophilic droplets within cells surrounding the secretory ducts and within palisade cells were detected, whereas the presence of polyphenols in the mesophyll was highlighted by toluide blue O. These observations have driven the subsequent phytochemical analyses. EO showed germacrene D (29.94%), carvacrol (14.3%) and eugenol (12.93%) as the most abundant compounds. In the HE, docosane, oleic and palmit acid, and lupeol were the predominant compounds, whereas caffeoylquinic acid and quercetin derivatives were the most common polyphenols in HAE. Considering the detected mean half-inhibitory concentrations (IC_50_), HAE showed predominant antioxidant activity (IC_50_ 30.54 µg/mL), while EO showed predominant anti-inflammatory activity (IC_50_ 397.59 µg/mL). Finally, HE, rich in medium-to-long fatty acids, showed the best protease inhibitory activity

## 1. Introduction

*Ptilostemon* Cass. is a little genus belonging to the tribe Cardue, subtribe Carduine of the Asteraceae family [1]. It is a peculiar component of the Mediterranean flora; in fact, its geographic distribution is restricted to the Mediterranean area, from Crimea and Turkey to the Iberian Peninsula and Morocco [2]. *Ptilostemon* consists of 15 species of annual or perennial herbs or shrubs, many of which are often narrow endemic [3]. *Ptilostemon greuteri* Raimondo & Domina is an endemic Sicilian shrub, which grows in a restricted area of Mt Inici (Municipality of Castellammare del Golfo, Trapani Province), in north-western Sicily [4]. It is a fruticose chamaephyte that can exceed 2 m in height, showing striated and grooved branches, white-tomentose at the apex. The leaves are persistent, soft, entire, lanceolate (20–30 × 2–3 cm), subsessile and sharp. They persist for a long time even after drying and are subglabrous and dark green in color on the upper surface, while the lower one is covered with a dense white tomentum. The flower heads are ovoid, 3–9 in number, arranged in a lax corymb, and show pink corollas [4]. At present, the species grows wild only in two sites of Mt Inici [5], and its survival is threatened by the frequent recurrence of summer fires [6]. According to Rivers [7], *P. greuteri* is classified as a Critically Endangered (CR) species.

*P. greuteri* is among the most interesting species of the Sicilian endemic vascular flora. Indeed, the morpho-anatomical characteristics of its leaves, and particularly their large surface area, appear to be a trait that strongly differs from that of the typical Mediterranean woody sclerophylls. Therefore, *P. greuteri* is considered a paleoendemic species, whose extreme rarity is presumably a consequence of the glacial and postglacial climate changes that occurred during the Quaternary [5]. Pasta et al. suggested that the persistency and survival of *P. greuteri* in Mt Inici could be related to local topographic and mesoclimatic factors, such as narrow and steep valleys representing microrefugia in arid environments [8].

Although the taxonomic and ecological aspects of this species have been thoroughly investigated [4,5,6,7,8], very few data exist about its anatomical and phytochemical characteristics.

Only one phytochemical study is today available on the aerial part of this species, collected during the flowering season [9]. The data obtained showed δ-cadinene, ß-cubebene and farnesol among the main components of the essential oil (EO). GC-MS analysis of the acetonic extract identified the presence of different triterpenes, while TLC analysis showed the presence of lignan and sesquiterpene lactones previously isolated also from other *Ptilostemon* species [9]. No data are available regarding any biological activity of this species.

On the contrary, recently, a hydroalcoholic extract of the related Sardinian species *Ptilostemon casabonae* (L.) Greuter has been studied from both a phytochemical and biological point of view, highlighting an interesting α-glucosidase-inhibitory activity [10,11,12].

Regarding the anatomical characteristics of *P. greuteri*, only some features of the woody stem have been investigated. In particular, the ring analysis, used to estimate the age of the largest plants, showed the presence of false growth rings probably in response to seasonal climate fluctuations [8]. On the contrary, no literature data regarding the anatomical and histological characteristics of the leaves of this species are today available.

Therefore, the aim of the present study was to investigate for the first time the anatomical and histochemical features of *P. greuteri* leaves searching to highlight the presence of specific compounds which could address the subsequent phytochemical and biological analyses of this paleoendemic species.

## 2. Results

### 2.1. Micro-Macro Morphological Analysis

**Figure 1:**
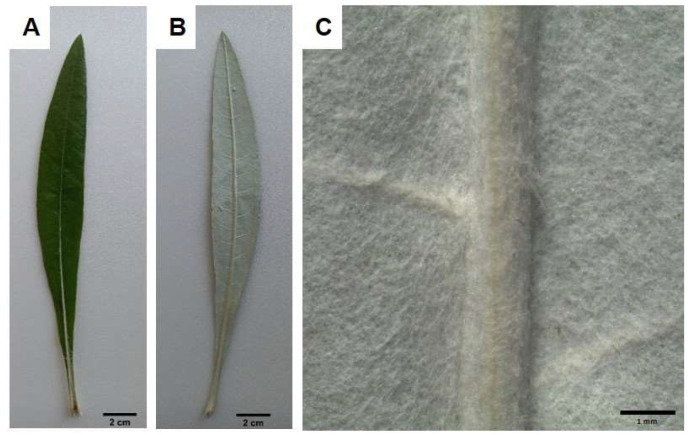
Macro-morphology of the leaf: (**A**) adaxial side; (**B**) abaxial side; (**C**) detail of the tomentum entirely covering the abaxial side.

Leaves had a large strongly lanceolate blade, dark green in color on the upper side (Figure 1A) and greyish on the lower one (Figure 1B). At high magnification, a dense tomentum entirely covering the abaxial surface of the leaf was detected (Figure 1C).

**Figure 2:**
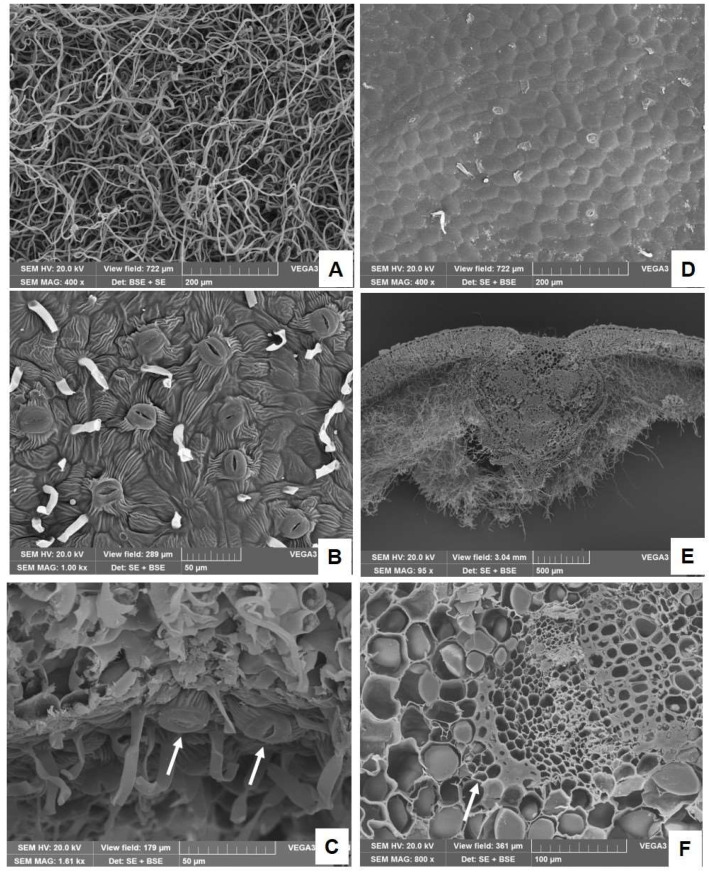
SEM micrographs of a leaf of *P. greuteri*: (**A**) non-glandular trichomes on the lower surface; (**B**) lower surface of the leaf from which non-glandular trichomes have been removed; stomata and striated cuticle are clearly visible; (**C**) enlargement of abaxial epidermis highlighting raised stomata (arrows); (**D**) upper glabrous surface of the leaf showing polygonal-shaped epidermal cells; (**E**) a transversal section of the leaf in which it can be observed the organization of vascular bundles inside the midrib and the dense tomentum on the lower epidermis; (**F**) magnification of a vascular bundle with a secretory duct near the phloem (arrow).

**Figure 3:**
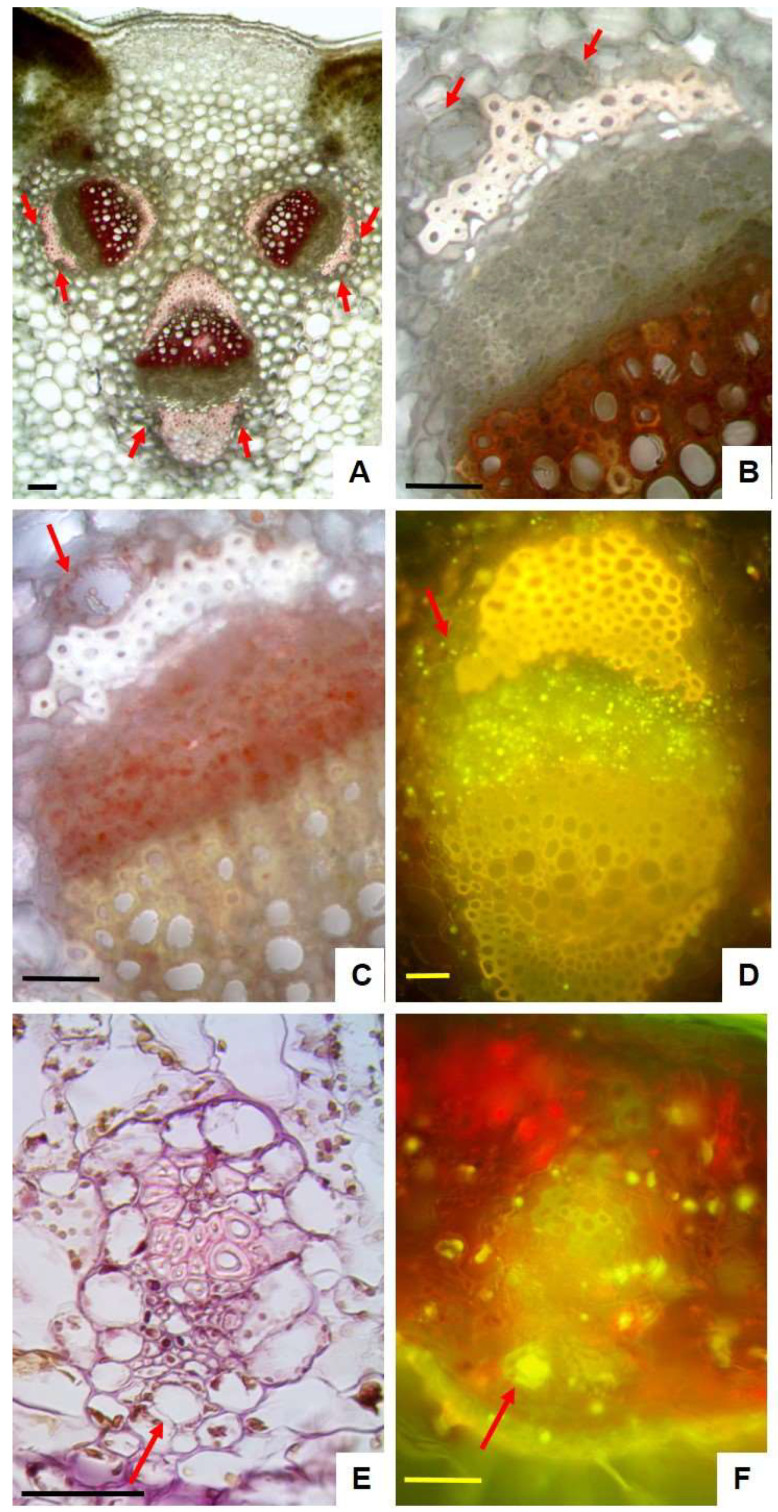
Leaf transversal sections of *P. greuteri*: (**A**,**B**) handmade sections stained with phloroglucinol and HCl: (**A**) detail of the midrib with three vascular bundles, secretory ducts (arrows) near the caps of supportive sclerenchyma stained pink with phloroglucinol and HCl; (**B**) magnification of a single vascular bundle in which two secretory ducts were clearly visible above the sclerenchyma overlying the phloem (arrows); (**C**,**D**) magnification of a vascular bundle in the midrib: stained with Sudan III (**C**) and Fluorol Yellow (**D**) highlighted the presence of small lipid droplets inside the secretory cells surrounding the ducts (arrow) and within the parenchyma cells of the phloem; (**E**,**F**) detail of the collateral vascular bundles in the mesophyll blade: (**E**) a semithin section stained with Hematoxylin–Eosin revealing the presence of one small secretory duct near the phloem (arrow); (**F**) a handmade section in which lipophilic substances inside the duct positively reacted with Fluorol Yellow (arrow). Bars: (**A**) = 100 µm; (**B**–**F**) = 50 µm.

Examination of the abaxial surface under the scanning electron microscope revealed that the tomentum was formed by a tangled layer of long and thin non-glandular trichomes (Figure 2A) that made it impossible to observe the epidermis beneath. The removal of non-glandular trichomes, by using a double-edged razor blade and tweezers, allowed highlighting the features of the epidermis, which was covered by a striated cuticle and showed many protruding stomata (Figure 2B,C, arrows). On the contrary, the adaxial epidermis was glabrous with cells polygonal in shape and covered by a layer of smooth cuticle (Figure 2D), and in transversal section it appeared uniseriate (Figure 2E). The leaves were hypostomatic with anomocytic—anisocytic type stomata, numerous on the abaxial one (Figure 2B), and on the contrary rare on the adaxial surface (Figure 2D). Moreover, the leaf was characterized by a dorsoventral structure, with the central rib protruding towards the abaxial surface and showing from one to five vascular bundles surrounded by a thick layer of collenchyma (Figure 2E and Figure 3A). On each side of vascular bundles two well-developed caps of supportive sclerenchyma were present and one to two small secretory channels were detected close to the sclerenchyma overlying the phloem (Figure 2F, arrow and Figure 3A,B, red arrows). When stained with lipid-specific dyes, small lipophilic droplets were detected in secretory cells surrounding the ducts (Figure 3C,D). In addition, they were also found within the parenchyma cells of the phloem, as demonstrated by their positive reaction with Sudan III (Figure 3C) and with Fluorol Yellow (Figure 3D). In the mesophyll blade, collateral vascular bundles were encircled by a parenchymatous bundle sheath with one small secretory duct close to the phloem (Figure 3E, arrow). The presence of lipophilic substances inside the ducts has been highlighted by Fluorol Yellow staining (Figure 3F, arrow).

**Figure 4:**
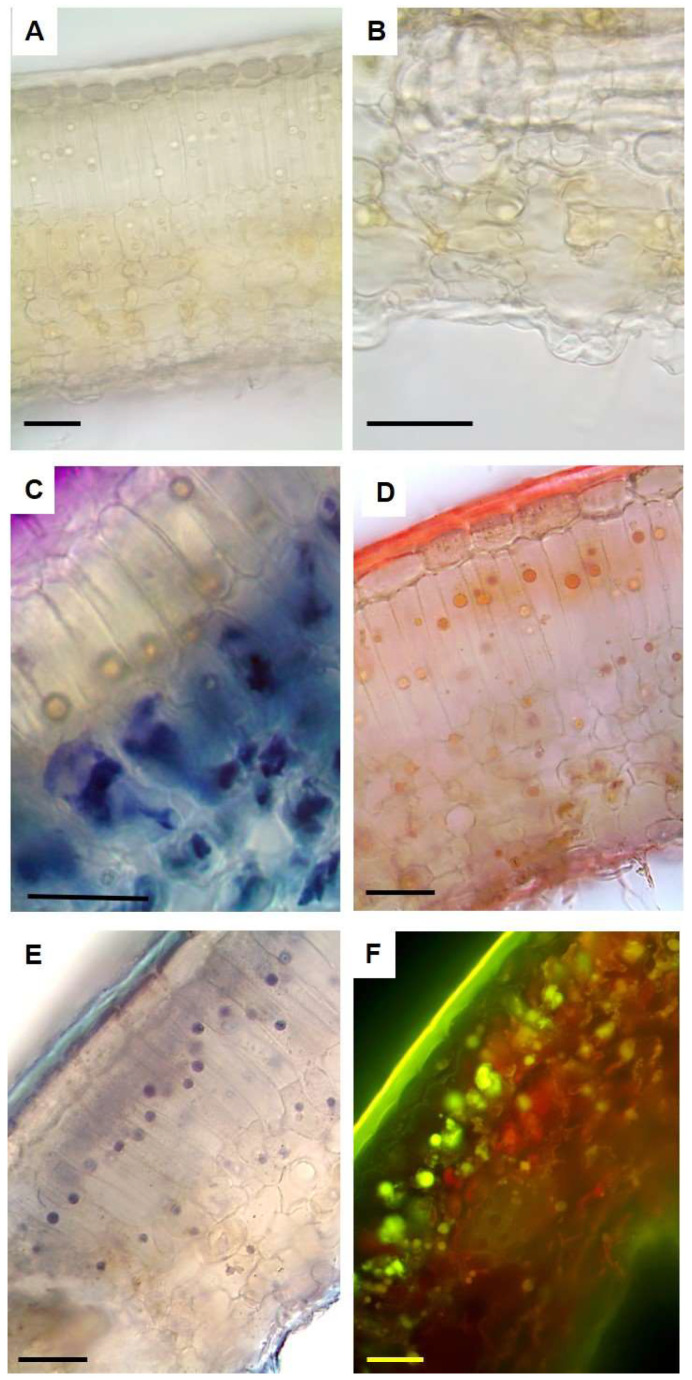
Handmade cross sections of leaves cleared with an aqueous solution of chloral hydrate (**A**–**E**) and then stained with (**C**) TBO, (**D**) Sudan III and (**E**) Sudan Black; (**F**) fresh handmade sections stained with Fluorol Yellow. (**A**) General anatomy evidencing the presence of single droplets in the palisade parenchyma cells; (**B**) detail of a protruding stomata on the lower epidermis; (**C**) droplets negatively reacted with TBO, while the presence of polyphenols was shown by the blue green staining; (**D**–**F**) droplets positively reacted with lipid-specific stains and stained orange with Sudan III (**D**), dark blue with Sudan Black (**E**) and bright yellow with Fluorol Yellow (**F**). Bars = 50 µm.

The clarification with an aqueous solution of chloral hydrate highlighted a bilayer mesophyll where the cells of the upper layer were more elongated than those of the innermost one (Figure 4A). On the adaxial side, a tick cuticle was visible in the cleared sections (Figure 4A) and it positively reacted with all lipid-specific dyes test (Figure 4D–F). The abaxial epidermis showed raised stomata (Figure 4B), as previously observed by SEM analysis (Figure 2C). The presence of a single spherical droplet per cell were observed in the palisade and more rarely in some cells of the spongy parenchyma (Figure 4A). In the bleached sections stained with TBO, no reaction of the droplets was observed, while the presence of polyphenols was shown by the blue-green staining (Figure 4C). On the contrary, the droplets reacted positively with lipid-specific dyes, appearing red/orange with Sudan III (Figure 4D), dark blue with Sudan Black (Figure 4E) and bright yellow with Fluorol Yellow (Figure 4F), highlighting their lipophilic nature.

### 2.2. Chemical Composition of Lipophilic Plant Complexes

The analysis of the essential oil composition, reported in Table 1, allowed the identification of 30 compounds, for a total of 99.98% of the total. Oxygenated monoterpenes are the main class of compounds (47.66%), followed by hydrocarbon sesquiterpenes (39.34%) and 10% of the compounds belong to chemical classes other than those previously mentioned. Finally, 2.12% belong to oxygenated sesquiterpenes. The main compound is germacrene D (29.94%), followed by carvacrol (14.3%) and eugenol (12.93%). Other compounds, whose quantity is between 5 and 10%, are thymol (7.96%) and linalool (6.07%). Finally, the following compounds are present in quantities greater than 1%: bicyclogermacrene (4.24%), docosane (3.35%), heneicosane (2.92%), β-caryophyllene (2.60%), nonanal (2.24%), α-terpineol (1.34%), verbenone (1.19%), τ-cadinol (1.15%) and α-humulene (1.01%).

In the hexane extract, 12 compounds were identified (99.12% of the total). The main compounds are docosane (20.75%), octadec-9-enoic acid (19.19%), hexadecanoic acid (16.92%) and lupeol (16.30%). The full composition is reported in Table 2.

### 2.3. Phytochemical Characterization of the Hydroalcoholic Extract

The phytochemical profile of the leaf hydroalcoholic extract was investigated by LC-DAD-ESI-MS analysis (Table 3). Compounds were detected and tentatively identified by comparison of mass and UV–Vis spectra with literature data and online free consulting spectra databases (SpectraBase^®^, PhytoHub, ReSpect for Phytochemicals, Mass Bank and PubChem), as well as with commercially available reference standards (see Table 3 footnotes for details).

**Figure 5:**
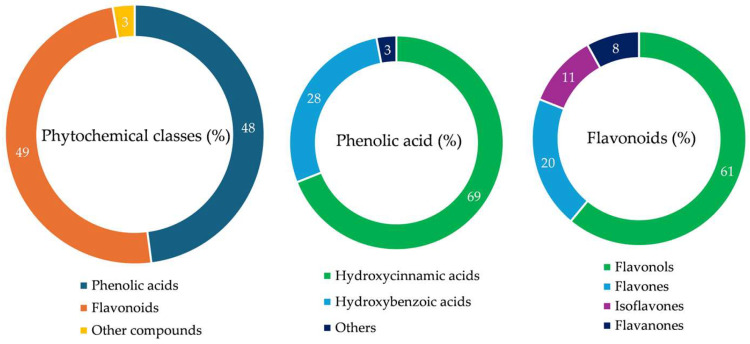
Phytochemicals identified in the leaf hydroalcoholic extract of *P. greuteri*. The results are expressed as a percentage of the main classes with respect to the total of the identified compounds. For phenolic acids and flavonoids, the subclasses to which they belong, with the relative percentages, were also identified.

Seventy-three compounds, belonging almost exclusively to the classes of flavonoids (49%) and phenolic acids (48%), were identified (Table 3). Examining the phytochemical profile in more detail, it can be noted that, among the phenol acids, the most numerous compounds are those belonging to the subclass of hydroxycinnamic acids (69%), with caffeoylquinic acid derivatives as the most representative compounds, followed by hydroxybenzoic acids (29%), particularly gallic, ellagic and benzoic acid derivatives (Table 3, Figure 5). Four flavonoid subclasses have been identified. The most numerous is certainly that of flavonols (64%), with quercetin, kaempferol and myricetin derivatives which represent the most present compounds. Among flavones (19%), apigenin and luteolin derivatives are those most frequently observed, as well as among isoflavones (11%), genistein and daidzein derivatives. Finally, the hesperetin derivatives are the most frequently observed flavanones (8%) (Table 3, Figure 5). The ellagitannin tellimagrandin I and the coumarin scopolin were also identified (Table 3).

### 2.4. Antioxidant and Anti-Inflammatory Properties

The antioxidant properties of the EO, HE and HAE against several charged radicals were investigated by several in vitro spectrophotometric and spectrofluorimetric assays based on different mechanisms and reaction environments. In particular, we evaluated the ferric reducing antioxidant power (FRAP), the scavenging activity against the 2,2-diphenyl-1-picrylhydrazyl radical (DPPH), the Trolox equivalent antioxidant capacity (TEAC) and oxygen reducing antioxidant capacity (ORAC). Furthermore, the ability of EO, HE and HAE to inhibit the heat-induced bovine serum albumin denaturation (ADA) and the protease inhibitory activity (PIA) were investigated to evaluate their anti-inflammatory properties.

Results, which were expressed as half-inhibitory concentration (IC_50_) with the respective confidence limits at 95%, are shown in Table 4.

As can be seen from Table 4, the behavior of the extracts varies greatly depending on the test used to evaluate the antioxidant and anti-inflammatory activity. However, what is clearly evident from the results is that HAE shows a predominantly antioxidant activity, while EO shows a predominantly anti-inflammatory activity. In fact, although EO shows lower IC_50_ values than HAE in the TEAC and ORAC tests, averaging the IC_50_ values observed in the four antioxidant tests, the average IC_50_ obtained for HAE is significantly lower than EO (30.54 vs. 53.46 µg/mL). The same can be said on the contrary for the anti-inflammatory activity, with EO showing a significantly lower average IC_50_ value than HAE (397.59 vs. 526.78 µg/mL). Surprisingly, HE, which showed the lowest antioxidant and anti-inflammatory activity, showed a very interesting IC_50_ in terms of protease inhibitory activity, which was not even statistically significant compared to the reference standard diclofenac sodium.

## 3. Discussion

*P. greuteri* is a rare plant endemic to Sicily considered as a “special” climate relict [8]. Several studies have examined the ecology of the species, highlighting how micro-environmental conditions, in particular water availability and land shape, are crucial in allowing the species to withstand the adverse surrounding climatic conditions. However, as also reported by Pasta et al. [8], there is a lack of data on the leaf anatomical features that may have favored the adaptive success of *P. greuteri*.

From a micromorphological point of view, some characteristics common to many drought-tolerant species adapted to the Mediterranean environment were observed.

*P. greuteri* leaf showed a thick layer of cuticle on the adaxial epidermis and a dense covering of non-glandular trichomes on the abaxial one. These trichomes give rise to a protective dense coating against drought and suitable for reducing the photoinhibition caused by high light intensity [13]. The thick layer of collenchyma, surrounding the vascular bundles of the central midrib, could be considered as an adaptation to a xeric environment [14]. In addition, the leaf blade was hypostomatic with a strongly developed palisade parenchyma, extended for more than half of the total mesophyll thickness, features that could be related to a better photosynthesis performance [15,16]. On the other hand, unlike xerophytic plants which show stomata sunken or at the same epidermal level, in this case raised stomata were found. This latter characteristic is generally common to mesophytes and could be related to an increase in the size of the substomatal chamber, facilitating gas exchange within the leaf, as previously suggested for *Begonia* by Papanatsiou et al. [17].

Differently from the Asteraceae family, which are characterized by different secretory structures [18,19,20,21], we did not find any glandular trichomes on the leaves. On the contrary, we observed little secretory ducts associated to vascular bundles both in the midrib and in the blade mesophyll. In agreement with data from some *Artemisia* species [21], one secretory duct for each vascular bundle of the blade mesophyll was found, appearing as a part of the parenchymal sheath. Instead, the midrib showed two ducts in proximity to the sclerenchyma cap covering the phloematic portion of the bundle. Therefore, they might be involved in the enhancement of nutrient transport, as suggested by Kromer et al. [18] in *Arnica* spp. Moreover, the occurrence in the midrib of several accessory vascular bundles was already previously observed in other Asteraceae [15,22,23] and it has been hypothesized that they are necessary to fulfil translocation requests during adverse environmental conditions [24].

Anatomical and histochemical analyses of the leaf also highlighted the presence of oil droplets or oil bodies in the mesophyll, predominantly in the palisade layer. This finding agrees with data from other Asteraceae, such as some *Arnica* and *Baccharis* species [18,19] and *Verbesina macrophylla* [20]. Oil bodies are specialized organelles commonly present in vegetative organs of many angiosperms, playing fundamental roles in metabolism, such as functions related to energy storage and to long distance transport of triglycerides. Recently, Benning et al. [25] suggested that they could be involved in transporting bound protein molecules in the phloem parenchyma to other tissues for storage and other uses [18,25]. In their study on the oil secretory system in different vegetative organs of three *Arnica* species, Kromer et al. [18] suggested that the triglycerides contained in the phloem parenchyma cells can become a substrate for the synthesis of the EO. In addition, it has been suggested that oil bodies may function to contrast different kind of stress by producing lipid compounds for plant defense responses [26].

The microscopic investigation carried out on *P. greuteri* leaves, in addition to providing details on the anatomical structure, allowed us to detect the presence of lipophilic droplets both within the cells surrounding the secretory ducts and palisade cells, as well as the presence of polyphenols in the mesophyll cells. Considering this, the study continued with the phytochemical characterization of the EO and the hexane extract (HE) to identify the main lipophilic compounds, and with the characterization of the hydroalcoholic extract (HAE) for the identification of the main polyphenolic compounds.

The EO profile of the *P. greuteri* leaves showed a phytochemical composition that completely disagrees with that reported in the only phytochemical study concerning this species [9]. Indeed, δ-cadinene (35.7%), β-cubebene (25.6%) and farnesol (10%) were previously detected as the most abundant compounds in the EO of the aerial parts collected during the flowering season. Among these, only δ-cadinene was identified in the present study, but with much lower relative abundance (0.31%). Camarda and collaborators had previously reported preliminary data on the phytochemical composition of the EO from aerial parts of *P. greuteri* collected in different vegetative phases [27]. Among the main constituents, in addition to eugenol, sesquiterpene compounds such as farnesol and farnesyl acetate were identified. This composition agrees partially with what was highlighted in the present study, eugenol being among the major components, but not the most abundant one (12.93%). On the contrary, farnesol and farnesyl acetate are absent.

Although there are no previous studies investigating the phytochemical profile of the HE of the aerial parts or specifically of the leaves of *P. greuteri*, Di Stefano and Pitonzo [9] investigated the phytochemical composition of an acetone extract of the *P. greuteri* aerial parts identifying, according to our results, β-amyrin and lupeol.

Regarding the leaf HAE, this is the first study which elucidated the whole polyphenolic profile of *P. greuteri* and, as such, a direct comparison with other literature data is not feasible. However, by studying the available literature data about the polyphenolic profile of other species belonging to the *Ptilostemon* genus, some interesting considerations can be made. Specifically, Sanna and collaborators [11] recently published a study concerning the antioxidant and antidiabetic properties of an ethanolic extract of *P. casabonae* leaves sampled in Sardinia. Although it is not the same plant species, what is immediately evident is how the two species have a similar polyphenolic profile, showing a marked presence of both phenolic acids and flavonoids. Also in this study, hydroxycinnamic acids and in particular caffeoylquinic acid derivatives are described as the most representative group of specialized metabolites, followed by quercetin, luteolin, kaempferol and apigenin derivatives. The results of this study also agree with a previous study conducted on the same plant species [10].

Polyphenolic compounds, in particular phenolic acids, flavonoids and coumarins, have been recognized to exert various beneficial effects on human health [28].

The powerful antioxidant activity of HAE, detected in the present study, is certainly attributable to the presence of numerous phenolic acids and flavonoids. It has been demonstrated that hydroxycinnamic acids and derivatives, the most common compounds detected in the present study, have shown a powerful and dose-dependent radical scavenging activity, higher also with respect to Trolox, generally used as a reference compound, as in the present study [29]. This activity is strictly dependent on their molecular structure, with the number and position of the phenols’ groups that play a pivotal role in the capacity to stabilize the resulting free radicals [29]. Moreover, the absence of a double bond in the side chain, as occurs in the hydrocaffeic series, gives rise to compounds with higher antioxidant activity, whereas methoxylation of the meta-phenolic group, such as in ferulic and hydroferulic derivatives, leads to a decrease in the antioxidant activity [29].

From a biological point of view, flavones are the most powerful flavonoids, followed by flavonols, flavanones and flavan-3-ols [30]. Glycosylation decreases their antioxidant properties, whereas the number and position of hydroxy groups increases it. In particular, the hydroxylation of the B ring plays a pivotal role, being one of the starting points of the oxidation chain. Finally, flavonoids can play a key role in lipid radical stabilization thanks to their hydrogen atoms’ transferability, making them less susceptible to autoxidation [30]. This highlighted structure–activity relationship can explain the powerful free-radical scavenging and anti-peroxidative activities detected for HAEs very rich in flavonols, such as quercetin, kaempferol and myricetin derivatives, and flavones such as apigenin and luteolin derivatives.

Current knowledge suggests that polyphenols can selectively inhibit several enzymes including pathological proteases [28]. Indeed, strong inhibitory effects of polyphenols on various serine proteases have been reported. Interestingly, it has been demonstrated that potent protease inhibition in micromolar range was displayed by rutin and derivatives esterified with medium- and long-chain, mono- and polyunsaturated fatty acids, followed by phloridzin and esculin esters with medium and long fatty acid chain length, while unmodified compounds showed only little or no effect. The authors suggested that the increased inhibitory properties of the acylated polyphenols may be related to their enhanced hydrophobicity [28]. In any case, medium to long fatty acids play a pivotal role in protease inhibitory activity, justifying what was observed in the present study with the HE, particularly richer in oleic and palmitic acid, with respect to HAE.

Regarding EO, the antioxidant activity of this plant-complex can be predominantly ascribed to the monoterpenes, especially those with strongly activated methylene groups. Among the oxygenated monoterpenes, the following order of potency can be postulated: phenolic monoterpenes > allylic alcohols > monoterpene aldehydes and ketones [31]. Although sesquiterpenes are thought to be compounds with poor antioxidant properties, oxygenated sesquiterpenes appear to exhibit a similar behavior to oxygenated monoterpenes [31]. Considering this, the antioxidant activity recorded in the present study can be mainly ascribed to carvacrol, thymol and linalool, followed by eugenol and germacrene D, although a potential synergistic effect cannot be excluded.

## 4. Materials and Methods

### 4.1. Plant Materials

**Figure 6:**
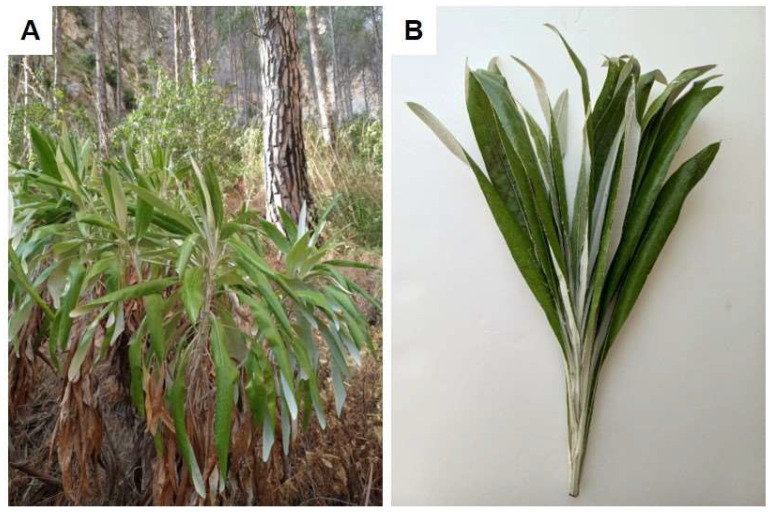
(**A**) *P. greuteri* growing at its collected site (Mt Inici); (**B**) detail of the leaves at the apex of a branch.

*P. greuteri* is a woody perennial tall shrub, showing leaves gathered at the apex of the branches (Figure 6A,B). In September 2023, at the end of the flowering period, 12–15 fully expanded leaves were collected from each plant, sampling a total of 30 adult plants. The plants grow at 323 m a.s.l., on the north-western limestone slopes of Mt Inici (38°01′08″ N, 12°52′08″ E), the classicus locus of the species, which falls within the municipal territory of Castellammare del Golfo (Trapani province, NW Sicily). From a bioclimatic point of view, this area is subject to the Mediterranean pluviseasonal oceanic bioclimate, with an upper Mediterranean thermotype and lower sub-humid ombrotype [32]. Voucher specimen with code 100084 has been deposited in the SAF herbarium of the Department of Agricultural, Food and Forest Sciences of the University of Palermo.

### 4.2. Light Microscope and Histochemical Analysis

The macro-morphological details of the leaves of *P. greuteri* were firstly investigated using a stereomicroscope (LEICA M205 C—Leica Microsystems, Wetzlar, Germany). Afterwards, fresh handmade cross sections, obtained by using a double-edged razor blade, were cleared with an aqueous solution of chloral hydrate and mounted in a chloral hydrate–glycerol solution [33]. For histochemical analyses the following staining was used: Phloroglucinol-HCl for lignin, TBO as general metachromatic stain and for polyphenols, and Sudan III, Sudan Black and Fluorol Yellow 088 for lipophilic substances [34]. Other leaves were fixed for 48 h in a FineFIX working solution (Milestone s.r.l., Bergamo, Italy), dehydrated in a series of reagent ethanol solutions (ranging from 70% to 100% ethanol in water) 1 h each [35] and paraffin embedded [36]. Ten-micron-thick cross sections were obtained using an automatic advanced rotative microtome (Leica RM 2255, Leica Biosystems, Heidelberg, Germany). After deparaffinization and rehydration, sections were stained with Hematoxylin–Eosin [34] for anatomical characterization. Observations were made with a Leica DM 2000 fluorescence microscope (Leica Microsystems, Wetzlar, Germany) using a ToupCam Digital Camera, CMOS Sensor 3.1 MP resolution (ToupTek Photonics, Hangzhou, China). For fluorescence staining, an H3 filter (excitation filter BP 420–490 nm) was used. Images were processed by ToupView software (version x64, 4.11.20805.20220506, ToupTek Photonics, Hangzhou, China).

### 4.3. Scanning Electron Microscopy

For a more detailed characterization of the leaf, also Scanning Electron Microscopy (SEM) analysis was carried out. Leaf small samples were handmade sectioned, then fixed and dehydrated as described above and subsequently critical point-dried using a K850CPD 2M (Strumenti S.r.l., Rome, Italy). The dried specimens were then free hand sectioned with a double-edged razor blade, mounted on aluminum stubs using two-sided adhesive carbon tape and covered with a 10 nm layer of gold particles [37]. The examination was performed using a VEGA3-Tescan-type LMU microscope (Tescan USA Inc., Cranberry Twp, PA, USA), operating at an accelerating voltage of 20 kV.

### 4.4. Extraction of the EO

Three kilograms of leaves were subjected to steam distillation, making the process last for 2 h, following the procedure reported in the European Pharmacopoeia [38]. The EOs obtained were subsequently solubilized in *n*-hexane and then filtered on anhydrous sodium sulphate and subjected to a flow of N_2_ to remove the residual solvent. Storage took place in amber vials, at +4 °C, away from sources of light, heat and humidity, until the time of testing and analysis. The following EO yields were obtained: 0.1% on dry weight, 0.003% on fresh weight.

### 4.5. GC/MS Analyses

The evaluation of the EO composition was performed by GC-MS analysis. An Agilent 6850 Ser apparatus (Agilent, Wilmington, DE, USA) was used, on which a DB-5 fused silica capillary column (30 m × 0.25 mm; film thickness 0.25 μm) was mounted. It was connected to an Agilent mass selective detector (MSD 5973) using the following parameters: ionization voltage 70 V; ion multiplier energy 2000 V. The mass spectra were scanned in the range of 40–500 *m*/*z*, with five scans per second, and the transfer line temperature was 295 °C. The analysis was programmed as follows: 5 min isothermally at 40 °C; then the temperature was increased by 2 °C/min up to 270 °C and finally it was held isothermally for 20 min. The analysis was also performed on an Innowax HP column (50 m × 0.20 nm; film thickness 0.25 μm) using the conditions reported above. In both cases, He was used as carrier gas (1.0 mL/min). The identification of the components was performed by comparing their Kovats indices (Ki), determined in relation to a homologous series of n-alkanes (C10–C35) under the same operating conditions, with those present in the literature [39,40,41,42] and by a careful comparison of the mass spectra with those of the pure compounds available in our laboratory or with those present in the NIST 17 and Wiley 257 mass libraries [43]. For some compounds, the identification was confirmed by co-injection with reference standards. The relative concentrations of the components were calculated by peak area normalization. Response factors were not considered.

### 4.6. Solvent Extraction of Non-Volatile Components

*P. greuteri* leaves (500 g and 150 g, rispectively) were extracted with 2 L n-hexane (HE) and 600 mL 70% ethanol (HAE), respectively, by maceration in glass flasks under constant stirring on a magnetic plate. The plant material was roughly reduced in size with scissors and placed in glass flasks in which the extraction solvent was then placed. The mixture was kept under constant stirring on a magnetic plate. An extraction cycle was made to last 5 days and in total 3 complete extraction cycles were carried out to maximize the extraction yield. The solvents of each cycle, containing the extract, were combined, filtered and subjected to the action of a rotary evaporator to remove the solvent and obtain the cleaned extract. The extracts weighed to calculate the extraction yields (3.01% and 2.25%, for HE and HAE, respectively). Finally, the extracts were stored in hermetically sealed tubes away from heat, light and humidity until subsequent analyses.

### 4.7. Determination of Fatty Acid Profile

To analyze the fatty acid composition, they were derivatized into fatty acid methyl esters (FAMEs), following a cold transmethylation procedure [44]. In an eppendorf, 2 mL of *n*-hexane were added to 100 mg of HE extract and the mixture was manually shaken for 2 s. To the mixture, 0.2 mL of methanolic potassium hydroxide solution (2 N) was added and the resulting mixture was transferred to a suitable test tube and centrifuged for 1 min (1400 rpm), before standing for 5 min. An amount of 975 μL of the upper phase, containing the FAMEs, was taken and transferred to 1.5 mL vials with 25 μL of external standard (nonadecanoate methyl ester 1000 ppm). Separation of FAMEs was performed using a Shimadzu gas chromatograph (GC-2010 Plus, Shimadzu, Kyoto, Japan) equipped with a flame ionization detector (FID). A fused silica capillary column (DB-wax; Agilent Technologies, Wilmington, DE, USA; length 30 m × id 0.25 mm and film thickness 0.25 μm) was used. Nitrogen was used as the carrier gas at a flow rate of 1.69 mL/min. The chromatographic gradient was as follows: the initial oven temperature was held at 165 °C for 15 min and then programmed to increase by 5 °C/min to 200 °C, held for 2 min, and followed by a second gradient of 5 °C/min to a final temperature of 240 °C, which was held for 5 min. The injector and detector temperatures were 250 and 280 °C, respectively. For the flame detector, hydrogen and compressed air were used. Finally, the injection volume was 1 μL with a split ratio of 50. The identification of the components was achieved by accurate analysis of the mass spectra.

### 4.8. Polyphenolic Profile Elucidation by LC-DAD-ESI-MS Analysis

The phytochemical profile of the HAE was investigated by LC-DAD-ESI-MS analysis by using an HPLC 1200 series coupled with an ion trap 6320 (Agilent technologies Inc., Santa Clara, CA, USA). Separation was carried out by a Luna Omega PS C18 column (150 mm × 2.1 mm, 5 μm; Phenomenex, Torrance, CA, USA) at 25 °C by using 0.1% formic acid (Solvent A) and acetonitrile (Solvent B) as a mobile phase according to the elution program reported in Danna et al. [45].

Injection volume and flow rate were set at 5 µL and 0.4 mL/min, respectively. UV–Vis spectra were recorded within the range 190–600 nm, whereas chromatograms were acquired at 260, 292, 330, 370, to detect all polyphenol classes. The ion trap acquisition following both positive and negative electrospray ionizaton (ESI) was set in full-scan mode (90–1000 *m*/*z*). Analytes identification was carried out by comparing the retention times, UV–Vis and MS spectra of each analyte with those of commercially available HPLC-grade reference standards (see Table 3), as well as with literature data and free online consulting UV–Vis and mass spectra databases (SpectraBase^®^, PhytoHub, ReSpect for Phytochemicals, Mass Bank and PubChem).

### 4.9. Antioxidant and Anti-Inflammatory Activity

The antioxidant and anti-inflammatory activity of *P. greuteri* EO, HE and HAE were evaluated by different in vitro spectrophotometric and spectrofluorimetric assays.

To guarantee the maximum solubility of the EO and the extracts under examination, 100 mg/mL stock solutions were prepared in DMSO for EO and HAE, and 7% random methylated β-cyclodextrin acetone/water (50:50, *v*/*v*) solution for HE, that were then properly diluted in the test solvent to assess the antioxidant and anti-inflammatory properties. All concentration ranges reported below refer to the final concentrations of EO, HE, HAE and reference compounds within the reaction mixture.

Results, which are the mean of three independent experiments in triplicate (*n* = 3), were expressed as inhibition (%) of the oxidative/inflammatory activity. The IC_50_ values (µg/mL) and the respective C.L. at 95% were calculated by Litchfield and Wilcoxon’s test (PHARM/PCS 4, MCS Consulting, Wynnewood, PA, USA). Trolox (0.25–20 µg/mL) and diclofenac sodium (3.0–40 µg/mL) were used as reference compounds for antioxidant and anti-inflammatory assays, respectively.

#### 4.9.1. FRAP Assay

The FRAP assay was carried out according to Ingegneri et al. [46]. Briefly, 10 μL of EO, HE and HAE (3.25–60.0 µg/mL, 125–2000 µg/mL and 1.875–30 μg/mL, respectively) were added to fresh, pre-warmed (37 °C) reagent (1:20, *v*/*v*) consisting of 300 mM buffer acetate (pH 3.6), 10 mM 2,4,6-Tris(2-pyridyl)-s-triazine dissolved in 40 mM hydrochloric acid, and 20 mM ferric chloride, and incubated for 4 min at RT in the dark. The absorbance was recorded at 593 nm using the Varioskan^TM^ LUX multimode microplate reader (Thermo Fischer Scientific, Waltham, MA, USA).

#### 4.9.2. DPPH Assay

The DPPH assay was carried out according to Ingegneri et al. [46]. Briefly, 3.75 μL of EO, HE and HAE (15–240.0 µg/mL, 30–480 µg/mL and 7.5–120 μg/mL, respectively) were added to fresh 6.3 mM DPPH methanol solution (1:40, *v*/*v*), mixed, and incubated in the dark for 20 min. The absorbance was recorded at 517 nm using the same instrument reported in Section 4.9.1.

#### 4.9.3. TEAC Assay

The TEAC assay was carried out according to Ingegneri et al. [46]. The radical reagent was prepared by mixing 1.7 mM 2,2′-azino-bis (3-ethylbenzothiazoline-6-sulfonic acid) with 4.3 mM potassium persulfate, and incubated in the dark for 12 h. Then, the radical solution was diluted in ethanol to obtain an average absorbance of 0.7 at 734 nm and used within 4 h. Ten microliters of EO, HE and HAE (3.75–60.0 µg/mL, 125–2000 µg/mL and 3.75–60 μg/mL, respectively) were added to the reagent (1:20, *v*/*v*) and incubated at RT for 6 min. The decrease in absorbance was recorded at 734 nm by using the same instrument reported in Section 4.9.1.

#### 4.9.4. ORAC Assay

The ORAC assay was carried out according to Ingegneri et al. [46]. Briefly, 20 μL of EO, HE and HAE (0.156–2.50 µg/mL, 1.875–30 µg/mL and 0.187–3.0 μg/mL, respectively) were added to fresh 117 nM fluorescein buffer solution (1:6, *v*/*v*), and incubated for 15 min at 37 °C. Then, 60 μL of 40 mM 2,2′-azobis(2-methylpropionamidine) dihydrochloride was added to trigger the reaction, which was recorded every 30 s for 90 min (λ_ex_ 485; λ_em_ 520) using the same instrument reported in Section 4.9.1.

#### 4.9.5. Albumin Denaturation Assay

The ability of *P. greuteri* plant complexes to inhibit heat-induced albumin denaturation was evaluated according to Cornara et al. [47]. Briefly, 0.4% fatty acid-free bovine serum albumin (BSA), phosphate buffer saline (pH 5.3) and sample (EO 50–800 µg/mL, HE 250–4000 µg/mL, and HAE 62.5–1000 µg/mL) were seeded in a 96-well plate (50:10:40, *v*/*v*/*v*) and the absorbance recorded immediately and after incubation for 30 min at 70 °C at 595 nm by using the same instrument reported in Section 4.9.1.

#### 4.9.6. Protease Inhibition Assay

The protease inhibitory activity was evaluated according to Cornara et al. [47]. Briefly, the sample (EO 25–400 µg/mL, HE 1.875–30 µg/mL and HAE 7.50–120.0 μg/mL) was added to a reaction mixture consisting of 10 μg/mL trypsin and 25 mM Tris-HCl buffer pH 7.5 (400 µL, 50:3:47, *v*/*v*/*v*). After this, 0.8% casein was added (200 µL) and the reaction mixture incubated for 20 min at 37 °C in a water bath. The reaction was stopped by adding 400 μL of perchloric acid. The cloudy suspension was centrifuged at 3500× *g* for 10 min, and the absorbance of the supernatant was recorded at 280 nm using a UV–Vis spectrophotometer (UV-1601, Shimadzu, Kyoto, Japan).

## 5. Conclusions

This is the first study which investigates, with a multidisciplinary approach, the leaves of *P. greuteri*, a rare Sicilian paleoendemic species. The pharmacognostic approach used has allowed us to highlight first the micro-morphological features, a useful tool for correct plant species identification. In addition, anatomical and histochemical analyses let us identify the presence of lipid droplets in secretory ducts as well as of oil bodies in the mesophyll; a rarely reported characteristic in the Asteraceae. These observations have driven the phytochemical analyses allowing to identify several lipophilic molecules, such as fatty acids and terpenes, correlated with the anti-inflammatory activity detected in the plant complexes. The polyphenols, histochemically and phytochemically detected by TBO and LC-DAD-ESI-MS analysis, respectively, were instead correlated mainly with the observed antioxidant activities. Finally, these investigations could also provide new research points to investigate the mechanisms involved in the drought tolerance of this rare paleoendemic species.

## Figures and Tables

**Table 1:** Phytochemical profile of the essential oil obtained from *P. greuteri* leaves.

N.	Compound	RT	%	Ki ^a^	Ki ^b^	Identification ^c^
1	Eucalyptol	20.07	0.61	952	1210	1, 2, 3
2	Linalool	25.608	6.07	1021	1543	1, 2, 3
3	Nonanal	25.844	2.24	1024	1392	1, 2
4	Camphor	28.198	0.76	1056	1491	1, 2, 3
5	Borneol	29.895	0.73	1079	1700	1, 2, 3
6	Terpinen-4-ol	30.762	0.72	1090	1601	1, 2, 3
7	α-Terpineol	31.816	1.34	1098	1662	1, 2, 3
8	Verbenone	32.931	1.19	1114	1720	1, 2
9	*cis*-Geraniol	36.524	0.54	1165	1839	1, 2
10	Thymol	39.373	7.96	1201	2164	1, 2, 3
11	Carvacrol	40.005	14.3	1210	2211	1, 2, 3
12	γ-Elemene	41.591	0.71	1234	1639	1, 2
13	Eugenol	43.264	12.93	1260	2163	1, 2, 3
14	*trans*-β-Damascenone	44.763	0.51	1282	1821	1, 2
15	Dodecanal	46.498	0.45	1303	1712	1, 2
16	β-Caryophyllene	46.696	2.6	1306	1598	1, 2
17	β-Copaene	47.328	0.53	1316	1580	1, 2
18	α-Humulene	48.79	1.01	1340	1667	1, 2
19	Germacrene D	50.661	29.94	1370	1708	1, 2
20	Bicyclogermacrene	51.478	4.24	1383	1734	1, 2
21	δ-Cadinene	53.176	0.31	1406	1756	1, 2
22	Spathulenol	56.199	0.41	1457	2127	1, 2
23	Hexadecane	57.847	0.3	1485		1, 2
24	τ-Cadinol	59.841	1.15	1513	2151	1, 2
25	α-Cadinol	60.585	0.56	1526	2227	1, 2
26	Diisobutyl phthalate	71.872	0.85	1727		1, 2
27	Palmitoleic acid	82.862	0.39	1945	2944	1, 2
28	Eicosane	99.365	0.36	2315		1, 2
29	Heneicosane	106.737	2.92	2491		1, 2
30	Docosane	113.601	3.35	2671		1, 2
	Total		99.98			
Monoterpene hydrocarbons			-			
Oxygenated monoterpenes			47.66			
Sesquiterpene hydrocarbons			39.34			
Oxygenated sesquiterpenes			2.12			
Others			10.86			

^a,b^ The Kovats retention indices are relative to a series of *n*-alkanes (C10–C35) on the apolar DB-5 and the polar HP Innowax capillary columns, respectively. ^c^ Identification method: 1 = comparison of the Kovats retention indices with published data, 2 = comparison of mass spectra with those listed in the NIST 02 and Wiley 275 libraries and with published data, and 3 = co-injection with authentic compounds.

**Table 2:** Composition of the *n*-hexane extract of *P. greuteri* leaves.

N.	Compound	%
1	Heneicosane	2.23
2	Docosane	20.75
3	Hexacosane	4.19
4	Hexadecanoic acid	16.92
5	Octadec-9-enoic acid	19.19
6	Octadecanoic acid	9.24
7	Nonadecanoic acid	5.30
8	Heicosanoic acid	1.12
9	Docosanoic acid	1.17
10	α-Tocopherol	0.24
11	β-Amyrin	2.47
12	Lupeol	16.30
	Total	99.12

**Table 3:** Phytochemical profile of *P. greuteri* hydroalcoholic extract (HAE) tentatively identified by LC-DAD-ESI-MS using both negative and positive ionization mode.

Compound Name	RT ^c^ (min)	Molecular Formula	Molecular Weight	[M−H]^−^ (*m*/*z*)	[M+H]^+^ (*m*/*z*)	Relative Abundance (%)	Peak n.
Benzoic acid-3-sulfate	3.4	C_7_H_6_O_6_S	218	—	219	1.84	1
Methoxyrosmarinic acid-sulfate	3.9	C_19_H_18_O_11_S	454	453	455	0.28	2
Galloyl glucose ^a^	4.2	C_13_H_16_O_10_	332	331	—	0.37	3
Quinic acid ^b^	5.0	C_7_H_12_O_6_	192	191	—	2.27	4
1,3-Dicaffeoylquinic acid ^a^	5.1	C_25_H_24_O_12_	516	—	517	2.40	5
Gallic acid 4-*O*-glucoside	5.3	C_13_H_16_O_10_	332	331	—	1.58	6
Coumaroylquinic acid	5.5	C_16_H_18_O_8_	338	—	339	3.74	7
Dihydroferulic acid-4′-sulfate	5.7	C_10_H_12_O_7_S	276	275	—	0.27	8
Protocatechuic acid-4-*O*-sulfate	6.1	C_7_H_6_O_7_S	234	233	—	0.22	9
Vanillic acid-4-*O*-sulfate	6.3	C_8_H_8_O_7_S	248	247	—	0.29	10
5-*O*-Caffeoylquinic acid (Chlorogenic acid) ^b^	6.6	C_16_H_18_O_9_	354	353	355	0.10	11
5-Feruloylquinic acid-4′-sulfate	8.6	C_17_H_20_O_12_S	448	447	—	0.04	12
3,5-Dihydroxybenzoic acid sulfate	14.2	C_7_H_6_O_7_S	234	233	—	0.03	13
3,4′-Dimethoxyrosmarinic acid-3′-sulfate	16.1	C_20_H_20_O_11_S	468	467	—	0.03	14
Diferulic acid	16.2	C_20_H_18_O_8_	386	—	387	0.32	15
1,5-Dicaffeoylquinic acid	17.0	C_25_H_24_O_12_	516	515	—	0.03	16
1,3-Dicoumaroylquinic acid	17.4	C_25_H_24_O_10_	484	483	—	0.04	17
3,5-Dicaffeoylquinic acid ^b^	17.8	C_25_H_24_O_12_	516	515	—	0.03	18
p-Coumaroyl-caffeoylquinic acid	20.8	C_25_H_24_O_11_	500	499	—	0.04	19
Caffeoyl-p-coumaroylquinic acid	21.2	C_25_H_24_O_11_	500	499	—	0.05	20
3,4′-Dimethoxyrosmarinic acid-4′-sulfate	21.6	C_20_H_20_O_11_S	468	—	469	0.46	21
1,5-Dicoumaroylquinic acid	22.4	C_25_H_24_O_10_	484	483	—	0.04	22
Isorhamnetin 3-*O*-[b-D-glucopyranosyl-(1->2)-a-L-rhamnopyranoside]	24.4	C_34_H_42_O_21_	624	623	—	0.07	23
3,3′-Dimethoxyrosmarinic acid-4′-sulfate	24.5	C_20_H_20_O_11_S	468	—	469	1.97	24
Succinyl caffeoyl-p-coumaroylquinic acid	27.1	C_29_H_29_O_15_	600	—	601	0.48	25
Apigenin 7-*O*-glucoside ^b^	27.5	C_21_H_20_O_10_	432	431	—	0.07	26
Succinyl-dicaffeoylquinic acid isomer	28.1	C_29_H_28_O_15_	616	615	617	0.05	27
4,5-dicaffeoylquinic acid	28.6	C_25_H_24_O_12_	516	515	—	0.04	28
Digallic acid	29.0	C_14_H_10_O_9_	322	—	323	0.51	29
Scopoletin 7-glucoside (Scopolin) ^a^	31.0	C_21_H_26_O_9_	354	353	355	0.55	30
Ellagic acid ^b^	31.3	C_14_H_6_O_8_	302	—	303	0.92	31
Genistein-7-*O*-glucuronide-4′sulfate	32.1	C_21_H_17_O_14_S	525	524	526	0.12	32
Succinyl-p-coumaroyl-caffeoylquinic acid	32.4	C_29_H_29_O_15_	600	599	—	0.07	33
Succinyl di-*O*-p-coumaroylquinic acid isomer	32.9	C_36_H_39_O_20_	584	583	585	0.11	34
Benzoic acid-3 glucuronide-4-sulfate	33.8	C_13_H_14_O_13_S	410	—	411	3.69	35
Ellagic acid glucoside	34.5	C_20_H_16_O_13_	464	—	465	0.85	36
Succinyl di-*O*-p-coumaroylquinic acid isomer	35.2	C_36_H_39_O_20_	584	583	585	0.38	37
Ferulic acid-5-5-caffeic acid	35.7	C_19_H_16_O_8_	372	371	373	2.97	38
Tellimagrandin I	36.0	C_34_H_26_O_22_	787	—	788	1.64	39
6,8-Dihydrokaempferol ^b^	37.4	C_15_H_12_O	318	—	319	1.09	40
Kaempferol 3-*O*-rutinoside ^b^	37.5	C_27_H_30_O_15_	594	593	—	0.11	41
Daidzein 4′-*O*-sulfate	38.0	C_15_H_10_O_7_S	334	—	335	1.12	42
Apigenin 7-*O*-(6″-malonyl-apiosyl-glucoside)	38.8	C_29_H_30_O_17_	650	649	—	0.17	43
Chrysoeriol 7-*O*-(6″-malonyl-glucoside)	39.2	C_25_H_24_O_14_	548	—	549	3.42	44
Kaempferol 3-*O*-(6″-malonyl-glucoside)	39.6	C_24_H_22_O_14_	534	533	535	0.48	45
Quercetin 3-*O*-arabinoside	39.9	C_20_H_18_O_11_	434	—	435	1.10	46
Succinyl-succinyl-dicaffeoylquinic acid	41.2	C_33_H_31_O_18_	716	715	717	0.11	47
Quercetin-3-(6″-malonyl)-glucoside	42.9	C_24_H_22_O_15_	550	549	551	0.48	48
Quercetin 3-*O*-rutinoside (Rutin) ^b^	43.5	C_27_H_30_O_16_	610	609	611	0.94	49
Quercetin 3-*O*-glucoside ^b^	45.1	C_21_H_20_O_12_	464	463	—	0.75	50
Quercetin-3-*O*-galactoside (Hyperoside) ^b^	46.5	C_21_H_20_O_12_	464	463	—	0.31	51
Quercetin 3-*O*-xylosyl-glucuronide	46.8	C_26_H_26_O_17_	610	609	—	0.11	52
Diosmetin ^b^	47.3	C_16_H_12_O_6_	300	—	301	6.31	53
6″-O-Malonylgenistin	47.8	C_24_H_22_O_13_	518	517	—	0.38	54
Luteolin 7-*O*-malonyl-glucoside	48.3	C_24_H_22_O_14_	534	533	535	1.04	55
Luteolin-3′-sulfate	49.0	C_24_H_22_O_14_	366	—	367	4.47	56
Myricetin 3-*O*-glucuronide	50.0	C_21_H_18_O_14_	494	493	495	1.39	57
Myricetin 5-*O*-glucuronide	50.4	C_21_H_18_O_14_	494	493	495	1.45	58
Quercetin disulfate	51.0	C_15_H_10_O_13_S_2_	462	—	463	1.57	59
Myricetin 3-*O*-glucoside ^b^	51.7	C_21_H_20_O_13_	480	479	—	0.23	60
Isorhamnetin 3-*O*-glucuronide	53.8	C_22_H_20_O_13_	492	—	493	7.75	61
Hesperetin-3′-sulfate-5,7-diglucuronide	53.9	C_28_H_30_O_20_S	719	718	—	0.90	62
Hesperetin-3′-glucuronide-5,7-sulfate	57.1	C_22_H_22_O_17_S_2_	622	621	—	0.09	63
Quercetin 3-*O*-rhamnosyl-rhamnosyl-glucoside	57.9	C_33_H_40_O_20_	756	755	—	0.08	64
Kaempferol 3-*O*-glucosyl-rhamnosyl-glucoside	60.6	C_33_H_40_O_20_	756	755	—	0.10	65
Kaempferol 3-*O*-rhamnoside^b^	63.3	C_21_H_19_O_10_	431	430	—	0.11	66
Quercetin ^b^	64.8	C_15_H_10_O_7_	302	—	303	4.55	67
Kaempferol 3-*O*-(2″-rhamnosyl-6″-acetyl-galactoside) 7-*O*-rhamnoside	65.1	C_29_H_32_O_16_	784	—	—	7.65	68
Lutein 7-*O*-glucuronide	68.1	C_21_H_18_O_12_	462	—	463	1.82	69
Luteolin ^b^	69.9	C_15_H_10_O_6_	286	—	287	5.75	70
Kaempferol 3-*O*-glucuronide	73.5	C_21_H_18_O_12_	462	—	463	4.06	71
Genistein 7-*O*-sulfate	81.8	C_21_H_18_O_14_S	350	—	351	4.96	72
Myricetin ^b^	82.7	C_15_H_10_O_8_	318	—	319	6.19	73

^a,b^ Checked with commercially available HPLC-grade reference standards (purity ≥ 98%) purchased from Extrasynthase (Genay, France) and Merck KGaA (Darmstadt, Germany), respectively; ^c^ RT, retention time; — not detected.

**Table 4:** Antioxidant and anti-inflammatory activity of *P. greuteri* leaf EO, HE and HAE, in comparison with the reference standards. Results are shown as mean of three independent experiments in triplicate (*n* = 3) and are expressed as the concentration inhibiting 50% of the oxidant/inflammatory activity (IC_50_) with 95% confidence limits (between brackets).

Test	EO	HE	HAE	RS ^a^
	µg/mL			
FRAP	25.54 (13.43–48.56) ^bc^	969.42 (592.36–1586.48) ^b^	24.83 (20.06–30.74) ^bc^	3.22 (1.26–8.19)
DPPH	166.68 (133.68–207.83) ^bc^	449.40 (359.14–562.36) ^b^	59.65 (48.46–73.43) ^bcd^	9.76 (5.71–16.70)
TEAC	20.86 (10.65–40.86) ^bc^	1490.04 (1059.43–2095.67) ^b^	36.63 (29.55–45.40) ^bcd^	6.35 (5.28–7.64)
ORAC	0.76 (0.50–1.13) ^c^	12.17 (5.29–28.01) ^b^	1.03 (0.43–2.47) ^bcd^	0.67 (0.27–1.64)
ADA	646.33 (496.50–841.38) ^bc^	2008.87 (1161.72–3473.77) ^b^	998.44 (770.75–1293.38) ^bcd^	23.52 (18.84–29.36)
PIA	151.85 (72.67–317.30) ^bc^	26.22 (20.75–33.15)	55.13 (31.29–97.14) ^bcd^	26.39 (15.26–45.63)

^a^ RS, Reference standard: Trolox for FRAP, DPPH, TEAC and ORAC assay; diclofenac sodium for anti-inflammatory assays (ADA and PIA); ^b^ *p* < 0.001 vs. RS; ^c^ *p* < 0.001 vs. HE; ^d^ *p* < 0.001 vs. EO.

## Data Availability

The original contributions presented in this study are included in the article. Further inquiries can be directed to the corresponding author.
